# Potential Risk Factors of Persistent Low Back Pain Developing from Mild Low Back Pain in Urban Japanese Workers

**DOI:** 10.1371/journal.pone.0093924

**Published:** 2014-04-08

**Authors:** Ko Matsudaira, Hiroaki Konishi, Kota Miyoshi, Tatsuya Isomura, Kyoko Inuzuka

**Affiliations:** 1 Clinical Research Center for Occupational Musculoskeletal Disorders, Kanto Rosai Hospital, Kawasaki, Kanagawa, Japan; 2 Department of Orthopaedic Surgery, Nagasaki Rosai Hospital, Sasebo, Nagasaki, Japan; 3 Spine Center, Yokohama Rosai Hospital, Yokohama, Kanagawa, Japan; 4 Clinical Research Department, CLINICAL STUDY SUPPORT, Inc., Nagoya, Aichi, Japan; University of Louisville, United States of America

## Abstract

**Study Design:**

Two-year, prospective cohort data from the Japan epidemiological research of occupation-related back pain study in urban settings were used for this analysis.

**Objective:**

To examine the association between aggravated low back pain and psychosocial factors among Japanese workers with mild low back pain.

**Summary of Background Data:**

Although psychosocial factors are strongly indicated as yellow flags of low back pain (LBP) leading to disability, the association between aggravated LBP and psychosocial factors has not been well assessed in Japanese workers.

**Methods:**

At baseline, 5,310 participants responded to a self-administered questionnaire including questions about individual characteristics, ergonomic work demands, and work-related psychosocial factors (response rate: 86.5%), with 3,811 respondents completing the 1-year follow-up questionnaire. The target outcome was aggravation of mild LBP into persistent LBP during the follow-up period. Incidence was calculated for the participants with mild LBP during the past year at baseline. Logistic regression was used to explore risk factors associated with persistent LBP.

**Results:**

Of 1,675 participants who had mild LBP during the preceding year, 43 (2.6%) developed persistent LBP during the follow-up year. Multivariate analyses adjusted for individual factors and an ergonomic factor found statistically significant or almost significant associations of the following psychosocial factors with persistent LBP: interpersonal stress at work [adjusted odds ratio (OR): 1.96 and 95% confidence interval (95%CI): 1.00–3.82], job satisfaction (OR: 2.34, 95%CI: 1.21–4.54), depression (OR: 1.92, 95%CI: 1.00–3.69), somatic symptoms (OR: 2.78, 95%CI: 1.44–5.40), support from supervisors (OR: 2.01, 95%CI: 1.05–3.85), previous sick-leave due to LBP (OR: 1.94, 95%CI: 0.98–3.86) and family history of LBP with disability (OR: 1.98, 95%CI: 1.04–3.78).

**Conclusions:**

Psychosocial factors are important risk factors for persistent LBP in urban Japanese workers. It may be necessary to take psychosocial factors into account, along with physical work demands, to reduce LBP related disability.

## Introduction

Low back pain (LBP) is a common musculoskeletal occupational health problem in industrialized countries and was found to be the leading specific cause of years lived with disability [1]. Japan is no exception, and LBP is one of the five most common health complaints of the Japanese general population [2]. Typically, 85–90% of the cases are classified as ‘non-specific’ [3], [4], and the majority of LBP is mild, so they do not become severely disabled [5], [6]. However, in terms of cost and work loss, the small proportion of people who become disabled due to LBP account for the largest occupational health care cost and the greatest number of work days lost around the world [7], [8]. Therefore, clarifying potential risk factors that could aggravate the LBP condition and lead to disability to work would be very important.

Many epidemiological studies of LBP have been conducted worldwide for decades. Psychosocial factors such as low job satisfaction, depression, or the tendency to somatize have been strongly indicated as ‘yellow flags’ for LBP leading to disability, as have ergonomic factors such as physical work demands [8]–[11], although the magnitude or intensity of each factor may vary across cultures or work environments [12]. Based on the above evidence, recently in Japan psychosocial factors began to be considered as a major risk for aggravating LBP. However, to our knowledge, the association between aggravation of Japanese workers' back pain and psychosocial factors has not been thoroughly assessed in prospective epidemiological research studies.

Previously, we reported potential risk factors for new onset of back pain disability in Japanese workers enrolled in a prospective cohort study in urban settings [13]. Data regarding various potential risk factors at baseline, as well as LBP-related outcomes, were collected prospectively. The cohort study focused mainly on LBP that caused work disability, a subject of critical importance to employers as well as workers, in terms of occupational health care.

The present study was designed to ascertain whether various psychosocial factors are associated with aggravating mild LBP into persistent LBP in workers with a 1-year history of mild LBP, using data from the previously reported cohort study; the findings of this further data analysis are reported here. This study was part of a series of clinical research projects conducted by the Japan Labor, Health and Welfare Organization related to 13 fields of occupational injuries and illnesses, including musculoskeletal disorders, mental health, and cancer. The research projects were conducted to help resolve occupational health issues and to disseminate the findings.

## Materials and Methods

### Data source

Data were extracted from a prospective cohort of the “The Japan epidemiological research of Occupation-related Back pain (JOB)” study. Participants were recruited from 16 workplaces in various occupational fields, located in or near Tokyo. The major occupational groups at these workplaces were office workers, nurses, sales/marketing personnel, and manufacturing engineers. Each participating organization was asked to distribute a self-administrated questionnaire to their workers, along with a cover letter from the study administration office. Respondents were asked to return their completed questionnaires by post, including their names and mailing addresses, which were used to send follow-up questionnaires directly from the study administration office. A total of 6,140 baseline questionnaires were distributed during September 2005 and February 2006, and 5,310 completed questionnaires were returned (response rate: 86.5%).

The baseline questionnaire included questions about the severity of the respondent's LBP and various individual and work-related factors. LBP severity was evaluated by the respondents themselves, who were asked to quantify the severity into one of four grades: grade 0, no LBP; grade 1, LBP not interfering with work; grade 2, LBP interfering with work; and grade 3, LBP interfering with work and leading to sick leave. The grades were determined with reference to Von Korff's grading method [14]. LBP was defined as pain localized between the costal margin and the inferior gluteal folds [3], and the area was depicted in the questionnaire. The baseline questionnaire included questions about the following: individual characteristics, including gender, age, obesity, smoking habits, history of LBP, and previous sick leave due to LBP; ergonomic work demands, such as frequency of bending, twisting or lifting at work; and psychosocial factors, such as depression, interpersonal stress at work, job control, job satisfaction, and somatization. A brief job stress questionnaire (BJSQ) was used to evaluate the major psychosocial factors [15], [16]. The BJSQ is a self-administered scale having a total of 57 items, developed by a research working group organized by the Japan Labour, Health and Welfare Organization. Question items for the questionnaire were extracted from standard questionnaires commonly used for evaluating stress related factors, psychological stress response, depression, anxiety, and somatization [17]–[23]. The questionnaire was assessed using standardized scores, which were classified into 19 work-related stress factors: mental workload (quantitative aspect), mental workload (qualitative aspect), physical workload, interpersonal stress at work, environmental work stress, job control, utilization of skills and expertise, physical fitness, job satisfaction, vigor, irritability, fatigue, anxiety, depression, somatic symptoms, support from supervisors, support from co-workers, support from family or friends, and daily-life satisfaction. For each factor above, standardized scores were developed on a 5-point scale ranging from 1 (lowest) to 5 (highest) based on a sample of more than 10,000 Japanese workers. The questionnaire has demonstrated moderate reliability, high internal consistency, and its criterion validity has been assessed with respect to the Job Content Questionnaire (JCQ) and The National Institute for Occupational Safety and Health (NIOSH) [24].

The follow-up questionnaire was distributed 1 year after the baseline questionnaire was administered. Of the 5,310 participants who completed the baseline questionnaire, 3,811 successfully completed and returned the follow-up questionnaire, resulting in a follow-up rate of 71.8%. The follow-up questionnaire included questions relating to LBP, such as severity of LBP during the past year, length of sick-leave due to LBP, whether medical care was sought, pain duration, and onset pattern. LBP severity was assessed by the respondents themselves, using the same categories as those of the baseline questionnaire.

Ethical approval for the study was provided by the review board of the Japan Labour, Health and Welfare Organization. Informed consent was obtained in writing from all participants.

### Data analysis

The outcome of interest was occurrence of persistent LBP during the 1-year follow-up period. In this study, persistent LBP was categorized as LBP interfering with work (grade 2 or grade 3), with disability lasting for longer than 3 months. Incidence was calculated for the participants who reported mild LBP (grade 1) during the past year at baseline. Participants were excluded from the analysis if they met any of the following criteria: a job change for reasons other than LBP; LBP due to a traffic accident; or LBP caused by a tumor, including metastasis, infection or fracture.

In addition to the compilation of simple, descriptive statistics, univariate and multivariate logistic regression analyses were used to explore risk factors associated with persistent LBP. Associations found by logistic regression analysis were summarized as odds ratios (ORs) with 95% confidence intervals (CIs). For the assessment of potential risk factors, crude ORs initially were estimated. Next, factors with P-values<0.1 were adjusted for individual factors, and also adjusted for individual factors and an ergonomic factor, in order to explore their potential risk factors. Factors with adjusted ORs that were statistically significant were considered to be potential risk factors. The following factors were used as adjusting factors because they are considered to be representative of individual and ergonomic factors: age, sex, obesity, smoking habits, education, and manual handling of objects [25]–[27]. Additionally, the above psychosocial risk factors were grouped by their correlations to explore multicollinearity, and then a statistically significant factor that had the highest adjusted ORs were selected from each group and applied to multivariate regression analysis. Statistical significance was assumed at the 5% level if the 95% CI did not overlap 1. All statistical calculations were carried out using the STATA 9.0 software package.

## Results

### Baseline characteristics of study participants

Of the 3,811 participants who responded to the 1-year follow-up questionnaires, 1,675 (excluding 43 who did not answer the question on LBP severity on their follow-up questionnaire) reported mild LBP during the past year at baseline and met the selection criteria. The mean age was 43.1 years (SD 10.1 years) and 1,342 (78.6%) were male. The mean BMI was 23.1 kg/m^2^ (SD 3.4 kg/m^2^). Of these participants, 1,165 (68.2%) were categorized as non-manual laborers; 147 (8.6%) as manual handlers of < 20-kg objects; 338 (19.8%) as manual handlers of ≥ 20-kg objects or as caregivers; and 58 (3.4%) were lacking job description data. In each category, the most common occupations were office work in the non-manual laborer category; manufacturing/engineering in the manual handler of < 20-kg objects category; and nurse in the manual handler of ≥ 20-kg objects or caregiver category.

The baseline characteristics of the 3,811 participants who provided follow-up data appeared to be not much different from those who did not. The mean (SD) ages were 42.9 (10.1) years and 38.0 (10.2) years, respectively, and the majority were male in both groups (80.6% and 82.8%, respectively). Those who completed the study had a mean (SD) BMI of 23.1 (3.3) while the values for dropouts were 22.9 (4.1). In the follow-up group (vs. the drop-out group), 78.6% (vs. 75.5%) were categorized as manually handling < 20-kg objects or not manually handling any objects in their work, 17.8% (vs. 18.9%) manually handled ≥ 20-kg objects or were working as caregivers, and data were lacking for 3.6% (vs. 5.6%). In both groups, the most common occupational fields in the categories of “manual handling of < 20-kg objects or not manually handling any objects”, and “manual handling of ≥ 20-kg objects or working as a caregiver” were office worker and nurse, respectively.

### Incidence of persistent LBP

Of the 1,675 eligible participants, 43 (2.6%) reported persistent LBP within the 1-year follow-up period. Of the 43 participants reporting persistent LBP, 76.7% had pain that persisted for longer than 6 months.

### Association between persistent LBP and potential risk factors

Crude ORs for persistent LBP, their 95% CIs, and P-values are shown in Table S1. The “somatic symptoms” risk factor was associated with an approximately 2.5-fold higher risk of suffering from persistent LBP. Associations of persistent LBP, with about a 2-fold risk increase, were also found with the following 5 psychosocial factors: interpersonal stress at work, job satisfaction, depression, support from supervisors, and daily-life satisfaction factors. An approximately 2-fold risk increase was found for the following 2 factors: previous sick-leave due to LBP and family history of LBP with work disability. Of the ergonomic factors, 7 (manual handling of objects at work, frequent bending, twisting, lifting, or pushing, hours of desk work, and physical workload) were associated with about a 3- to 4-fold higher risk of developing persistent LBP. These 15 factors were chosen for multivariate logistic regressions, and the results are shown in Table 1. Most of the ergonomic factors were significant with the ORs adjusted for individual factors. Five factors from the BJSQ (interpersonal stress at work, job satisfaction, depression, somatic symptoms, and support from supervisors), as well as previous sick-leave due to LBP and family history of LBP with disability, remained statistically significant or almost significant by adjusted ORs. The magnitudes of adjusted ORs of these factors did not markedly change from our crude OR analyses. Among the 5 factors from the BJSQ, interpersonal stress at work, job satisfaction, and support from supervisors tended to correlate to each other, and depression and somatic symptoms tended to correlate to each other (Spearman's rho, data not shown). Additional multivariate regression analysis included job satisfaction and somatic symptoms from the BJSQ psychosocial factors and family history of LBP with disability, chosen by the statistical significance of the adjusted OR. As shown in Table 2, all of the factors remained statistically significant or almost significant in the multivariate analysis.

**Table 1:** Adjusted odds ratios of the baseline factors for persistent low back pain (LBP) with work disability; factors with crude odds ratio P values<0.1.

Factors		%	OR Adjusted for individual factors^a^		OR Adjusted for individual factors and an ergonomic factor^b^	
			OR	95%CI	OR	95%CI
Previous sick leave due to LBP	No previous sick leave	76.5	1.00		1.00	
	Previous sick leave	23.5	1.92	0.99–3.74	1.94	0.98–3.86
Manual handling of materials at work	Manual handling of < 20-kg objects including desk work	79.5	1.00			
	Manual handling of ≥ 20-kg objects or working as a caregiver	20.5	2.70	1.98–8.67	-	-
Bending^c^	Infrequent	88.7	1.00			
	Frequent	11.3	3.45	1.54–7.72	-	-
Twisting^c^	Infrequent	94.6	1.00			
	Frequent	5.4	4.35	1.80–10.52	-	-
Lifting^c^	Infrequent	89.6	1.00			
	Frequent	10.4	2.81	1.18–6.66	-	-
Pushing^c^	Infrequent	95.2	1.00			
	Frequent	4.8	3.48	1.24–9.76	-	-
Hours of desk work^d^	< 6 hours per day	53.9	1.00		1.00	
	≥ 6 hours per day	46.1	0.45	0.23–0.88	0.66	0.31–1.40
Physical workload^e^	No stress	61.9	1.00		1.00	
	Stress	38.1	2.22	1.16–4.23	1.53	0.70–3.33
Interpersonal stress at work^e^	No stress	78.8	1.00		1.00	
	Stress	21.2	2.04	1.06–3.93	1.96	1.00–3.82
Job satisfaction^e^	Satisfied	77.3	1.00		1.00	
	Not satisfied	22.7	2.48	1.31–4.70	2.34	1.21–4.54
Depression^e^	Not feeling depressed	64.6	1.00		1.00	
	Depressed	35.4	2.09	1.10–3.99	1.92	1.00–3.69
Somatic symptoms^e^	No somatic symptoms	63.4	1.00		1.00	
	Somatic symptoms	36.6	2.99	1.55–5.75	2.78	1.44–5.40
Support from supervisors^e^	Support	74.0	1.00		1.00	
	No support	26.0	1.97	1.04–3.73	2.01	1.05–3.85
Daily-life satisfaction^e^	Satisfied	68.7	1.00		1.00	
	Not satisfied	31.3	1.81	0.97–3.40	1.61	0.84–3.08
Family history of LBP with disability	No LBP with disability	74.6	1.00		1.00	
	LBP with disability	25.4	2.02	1.07–3.81	1.98	1.04–3.78

OR: odds ratio, CI: confidence interval, LBP: low back pain

a Adjusted for age, gender, obesity, smoking habits, and education.

b Adjusted for age, gender, obesity, smoking habits, education, and manual handling of materials at work.

c Bending, twisting, lifting, and pushing: ≥ half of the day was considered frequent.

d Hours of desk work: longer than 6 hours per day was considered to be static posture.

e Work-related stress factors assessed with the brief job stress questionnaire: not feeling stressed, feeling stressed: the 5 original responses were reclassified into “not feeling stressed”, where low, slightly low and moderate were combined, and “feeling stressed”, where slightly high and high were combined.

**Table 2:** Multivariate-adjusted odds ratios for the persistent low back pain (LBP).

Factors		Adjusted OR^a^	95%CI	P value
Job satisfaction	Satisfied	1.00		
	Not satisfied	2.03	1.01–4.07	0.046
Somatic symptoms	No somatic symptoms	1.00		
	Somatic symptoms	2.46	1.25–4.83	0.009
Family history of LBP with disability	No LBP with disability	1.00		
	LBP with disability	2.00	1.03–3.88	0.042

OR: odds ratio, CI: confidence interval, LBP: low back pain.

a Adjusted for individual factors (age, gender, obesity, smoking habits, and education) and an ergonomic factor (manual handling).

## Discussion

Potential risk factors for people with LBP that could aggravate the condition and cause too much disability to work were explored in a cohort of urban Japanese workers. The incidence of persistent LBP developing from mild LBP was 2.6%. ORs adjusted for individual factors and an ergonomic factor (manual handling of objects) showed that low job satisfaction, lack of support from supervisors, interpersonal stress at work, depression, somatic symptoms, and a family history of LBP with disability were significant risk factors, and previous sick leave a nearly significant risk factor, for development of persistent from mild LBP. Our results indicate that these psychosocial factors are important in urban Japanese workers who have made the transition from mild to persistent LBP.

In this study, the definition of persistent LBP was disability longer than 3 months, and the index for disability was LBP interfering with work, with or without sick leave. In Western countries, ‘absence from work' is often used as an outcome measurement for disability. The number of participants who were absent due to LBP (grade 3) was relatively small. Our previous international epidemiological study showed that taking sick leave due to musculoskeletal disorders, mostly LBP, appears to be less common among Japanese workers than British workers [28]. The lower percentage of absence due to LBP in Japanese workers compared to workers in European countries may be due to a difference in concerns about being absent, such as worries that it might affect employment, salary increases, or evaluations of work performance. In fact, the proportion of Japanese workers with disability irrespective of taking sick leave (sick leave defined as any unplanned absence from work) was approximately the same as the proportion of UK workers with sickness-related absences. Additionally, in another international cross-sectional study, the prevalence of disabling LBP varied markedly across countries, and the Japanese workers showed the lower prevalence than in other countries [29]. Therefore, when assessing Japanese workers, it seems appropriate to define LBP disability as LBP interfering with work, with or without sick leave.

Among the five factors from the BJSQ (low job satisfaction, little support from supervisors, interpersonal stress at work, depression, and somatic symptoms), low job satisfaction, little support from supervisors, and interpersonal stress at work tend to relate to each other, and depression and somatic symptoms tend to relate to each other. The first three factors (e.g., low job satisfaction) could be considered stressful conditions that directly and negatively affect the individual, and the latter two factors (e.g., depression) as symptoms of both physical and mental stress. Generally, the symptoms of somatization are headaches, neck and shoulder discomfort, dizziness, palpitations or shortness of breath, diarrhea or constipation, and back pain, and these symptoms are triggered by emotional discomfort and psychosocial distress [30]. Individuals with somatization often complain of pain in various locations, functional disturbance of various organ systems, and are depressed or overwhelmed by these symptoms. Patients falling into such a situation are usually said to suffer from functional somatic syndrome (FSS) [31], [32]. Our results could suggest that workers with mild LBP, under frazzled, depressed, or somatizing conditions, accompanied by emotional discomfort and psychosocial distress (e.g., low job satisfaction, little social support from supervisors, and interpersonal stress at work), did not manifest disabling back pain as a symptom of FSS at baseline, but the pain became disabling during the following year.

A family history of persistent LBP was also suggested as a psychosocial risk factor in this analysis. Second-hand experience of LBP among people with whom a worker is in very close contact (families, friends, or partners) may make it easier to imagine how mild LBP transforms to persistent LBP. Previous research has revealed that some people can share another person's physical pain experience, in both emotional and sensory components, by just observing the other person's pain [33], [34]. Family members, therefore, may provide reinforcement for sick behavior [35], even though these family members do not have had any disorders, such as back pain [36]–[39].

Psychosocial intervention has been reported to improve overall well-being, as well as reducing distress and physical complaints, in patients with LBP in Western countries [40]. This intervention is based on the hypothesis that psychosocial factors are associated with the transition to persistent LBP, and should be examined in future research studies in Japan.

Limitations of the current study should be mentioned. One is the fact that the majority of the subjects were males, and that a broad range of Japanese occupations was not represented. The study cohort was not a representative sample of the entire Japanese workers in urban areas; therefore, the generalizability of the findings may be limited. Secondly, although cognitive and emotional aspects of back pain are known to influence disability aggravation, some important psychosocial factors, such as the attitudes of health care providers, and catastrophizing and fear-avoidance beliefs, were not included in this analysis. This was because appropriate questionnaires were not available in the Japanese language. Future studies should include additional self-reported outcome measures, such as results of the Fear-Avoidance Belief Questionnaire (FABQ) [41], [42] or the Tampa Scale of Kinesiophobia (TSK) [43], [44], to assess the impact of these factors in Japanese workers. The Japanese versions of these questionnaires are now being developed.

Psychosocial factors are one of the most important risk factors for making the transition to persistent LBP from mild LBP in urban Japanese workers. In the future, preventive strategies for reducing persistent LBP in the workplace should deal not only with physical work demands, which is already well-understood, but potentially should incorporate psychosocial management techniques as well.

## Supporting Information

Table S1 **Crude odds ratios of the baseline factors for persistent low back pain (LBP) with work disability.** OR: odds ratio, CI: confidence interval, BMI: body mass index, LBP: low back pain. ^a^ Obesity: BMI of ≥ 25 is defined as obesity in Japan. ^b^ Smoking habits: Brinkmann index of ≥ 400 was defined as heavy smoker, calculated from the total number of cigarettes smoked per day multiplied by duration of smoking in years [45]. ^c^ Working hours: ≥ 60 hours per week was assumed to be uncontrolled overtime. ^d^ Bending, twisting, lifting, and pushing: ≥ half of the day was considered frequent. ^e^ Hours of desk work: longer than 6 hours per day was considered as static posture. ^f^ Work-related stress factors assessed with the brief job stress questionnaire: not feeling stressed, feeling stressed: the 5 original responses were reclassified into “not feeling stressed”, where low, slightly low and moderate were combined, and “feeling stressed”, where slightly high and high were combined. ^g^ Monotonous task: feelings of monotony or boredom at work.
(DOC)
