# Supplementary material for: Potential Risk Factors of Persistent Low Back Pain Developing from Mild Low Back Pain in Urban Japanese Workers
Source: PLoS One. 2014 Apr 8;9(4):e93924. doi: 10.1371/journal.pone.0093924 (PMC3979726; doi:10.1371/journal.pone.0093924)
Supplement: Table S1 — Crude odds ratios of the baseline factors for persistent low back pain (LBP) with work disability. OR: odds ratio, CI: confidence interval, BMI: body mass index, LBP: low back pain. a Obesity: BMI of ≥ 25 is defined as obesity in Japan. b Smoking habits: Brinkmann index of ≥ 400 was defined as heavy smoker, calculated from the total number of cigarettes smoked per day multiplied by duration of smoking in years [45]. c Working hours: ≥ 60 hours per week was assumed to be uncontrolled overtime. d Bending, twisting, lifting, and pushing: ≥ half of the day was considered frequent. e Hours of desk work: longer than 6 hours per day was considered as static posture. f Work-related stress factors assessed with the brief job stress questionnaire: not feeling stressed, feeling stressed: the 5 original responses were reclassified into “not feeling stressed”, where low, slightly low and moderate were combined, and “feeling stressed”, where slightly high and high were combined. g Monotonous task: feelings of monotony or boredom at work. (DOC) [file pone.0093924.s001.doc]

| Factors |  | % | Crude OR | 95%CI | P value |
| --- | --- | --- | --- | --- | --- |
|
| Age (Years) | < 40 | 38.4 | 1.00 |  |  |
|  | 40-49 | 32.5 | 0.94 | 0.42–2.09 | 0.885 |
|  | ≥ 50 | 29.2 | 1.75 | 0.86–3.56 | 0.120 |
| Gender | Male | 78.6 | 1.00 |  |  |
|  | Female | 21.4 | 0.83 | 0.38–1.80 | 0.634 |
| Obesity ^a^ | < BMI 25 kg/m^2^ | 74.7 | 1.00 |  |  |
|  | ≥ BMI 25 kg/m^2^ (obesity) | 25.3 | 1.15 | 0.58–2.25 | 0.691 |
| Smoking habits ^b^ | Non-smoker or light smoker | 70.2 | 1.00 |  |  |
|  | Heavy smoker | 29.8 | 1.49 | 0.79–2.81 | 0.216 |
| Education | College/University | 72.4 | 1.00 |  |  |
|  | High school/Junior high school | 27.6 | 1.14 | 0.59–2.21 | 0.688 |
| Length of employment at current job | < 5 years | 32.4 | 1.00 |  |  |
|  | ≥ 5 years | 67.6 | 1.84 | 0.88–3.87 | 0.106 |
| Working hours per week ^c^ | < 60 hours | 84.4 | 1.00 |  |  |
|  | ≥ 60 hours | 15.6 | 0.88 | 0.37–2.09 | 0.764 |
| Work shift | Day shift | 77.0 | 1.00 |  |  |
|  | Night shift | 23.0 | 1.63 | 0.85–3.12 | 0.140 |
| Previous sick leave due to LBP | No previous sick leave | 76.5 | 1.00 |  |  |
|  | Previous sick leave | 23.5 | 2.19 | 1.18–4.09 | 0.013 |
| Manual handling of objects at work | Manual handling of < 20-kg objects including desk work | 79.5 | 1.00 |  |  |
|  | Manual handling of ≥ 20-kg objects or working as a caregiver | 23.5 | 2.70 | 1.44–5.07 | 0.002 |
| Bending ^d^ | Infrequent | 88.7 | 1.00 |  |  |
|  | Frequent | 11.3 | 2.58 | 1.25–5.34 | 0.011 |
| Twisting ^d^ | Infrequent | 94.6 | 1.00 |  |  |
|  | Frequent | 5.4 | 3.75 | 1.62–8.68 | 0.002 |
| Lifting ^d^ | Infrequent | 89.6 | 1.00 |  |  |
|  | Frequent | 10.4 | 2.03 | 0.93–4.45 | 0.077 |
| Pushing ^d^ | Infrequent | 95.2 | 1.00 |  |  |
|  | Frequent | 4.8 | 2.67 | 1.02–6.98 | 0.045 |
| Hours of desk work ^e^ | < 6 hours per day | 53.9 | 1.00 |  |  |
|  | ≥ 6 hours per day | 46.1 | 0.50 | 0.26–0.96 | 0.039 |
| Mental workload (quantitative aspect) ^f^ | No stress | 54.9 | 1.00 |  |  |
|  | Stress | 45.1 | 0.65 | 0.34–1.22 | 0.182 |
| Mental workload (qualitative aspect) ^f^ | No stress | 56.3 | 1.00 |  |  |
|  | Stress | 43.7 | 0.92 | 0.50–1.70 | 0.791 |
| Physical workload ^f^ | No stress | 61.9 | 1.00 |  |  |
|  | Stress | 38.1 | 1.72 | 0.94–3.15 | 0.080 |
| Environmental work stress ^f^ | No stress | 73.7 | 1.00 |  |  |
|  | Stress | 26.3 | 0.96 | 0.48–1.93 | 0.919 |
| Job control ^f^ | Controlled | 35.2 | 1.00 |  |  |
|  | Not controlled | 64.8 | 0.79 | 0.42–1.48 | 0.464 |
| Utilization of skills and expertise ^f^ | Utilization of skills and expertise | 84.3 | 1.00 |  |  |
|  | No utilization of skills and expertise | 15.7 | 0.87 | 0.37–2.09 | 0.762 |
| Physical fitness ^f^ | Feeling fit | 75.8 | 1.00 |  |  |
|  | Not feeling fit | 24.3 | 1.37 | 0.71–2.64 | 0.356 |
| Job satisfaction ^f^ | Satisfied | 77.3 | 1.00 |  |  |
|  | Not satisfied | 22.7 | 2.52 | 1.36–4.68 | 0.003 |
| Vigor ^f^ | Vigorous | 81.5 | 1.00 |  |  |
|  | Not vigorous | 18.6 | 1.74 | 0.88–3.42 | 0.111 |
| Anger ^f^ | Not angry | 65.4 | 1.00 |  |  |
|  | Angry | 34.8 | 1.37 | 0.74–2.53 | 0.319 |
| Fatigue ^f^ | No fatigue | 62.6 | 1.00 |  |  |
|  | Fatigue | 37.4 | 1.61 | 0.88–2.96 | 0.122 |
| Anxiety ^f^ | Not anxious | 69.8 | 1.00 |  |  |
|  | Anxious | 30.2 | 1.13 | 0.59–2.15 | 0.715 |
| Depression ^f^ | Not feeling depressed | 64.6 | 1.00 |  |  |
|  | Depressed | 35.4 | 1.77 | 0.95–3.29 | 0.072 |
| Somatic symptoms ^f^ | No somatic symptoms | 63.4 | 1.00 |  |  |
|  | Somatic symptoms | 36.6 | 2.51 | 1.34–4.71 | 0.004 |
| Support from supervisors ^f^ | Support | 74.0 | 1.00 |  |  |
|  | No support | 26.0 | 1.89 | 1.01–3.51 | 0.045 |
| Support from coworkers ^f^ | Support | 62.3 | 1.00 |  |  |
|  | No support | 37.7 | 1.19 | 0.65–2.21 | 0.571 |
| Support from family or friends ^f^ | Support | 77.8 | 1.00 |  |  |
|  | No support | 22.2 | 0.85 | 0.39–1.86 | 0.686 |
| Daily-life satisfaction ^f^ | Satisfied | 68.7 | 1.00 |  |  |
|  | Not satisfied | 31.3 | 1.78 | 0.97–3.28 | 0.064 |
| Monotonous work ^g^ | Not monotonous | 83.4 | 1.00 |  |  |
|  | Monotonous | 16.6 | 0.97 | 0.43–2.19 | 0.932 |
| Family history of LBP with disability | No LBP with disability | 74.6 | 1.00 |  |  |
|  | LBP with disability | 25.4 | 1.96 | 1.05–3.64 | 0.035 |
